# Free Fatty Acid Species Differentially Modulate the Inflammatory Gene Response in Primary Human Skeletal Myoblasts

**DOI:** 10.3390/biology10121318

**Published:** 2021-12-12

**Authors:** Melanie Rauen, Dandan Hao, Aline Müller, Eva Mückter, Leo Cornelius Bollheimer, Mahtab Nourbakhsh

**Affiliations:** Department of Geriatric Medicine, RWTH Aachen University Hospital, 52074 Aachen, Germany; melanie.rauen@rwth-aachen.de (M.R.); dhao@ukaachen.de (D.H.); almueller@ukaachen.de (A.M.); emueckter@ukaachen.de (E.M.); cbollheimer@ukaachen.de (L.C.B.)

**Keywords:** human primary skeletal muscle cells, free fatty acids, inflammation, receptor tyrosine kinases, phosphorylation pathways, cytokines, chemokines

## Abstract

**Simple Summary:**

Epidemiological studies show that obesity increases the risk of muscle mass loss with age, a syndrome called sarcopenic obesity. Obesity leads to increased free fatty acids (FFAs) and excessive fat deposits, which impair the integrity of skeletal muscles by unknown mechanisms. This report indicates that FFAs directly affect human skeletal muscle cell replication and inflammatory gene expression. The structural characteristics of FFAs play a decisive role in triggering both processes. Thus, the characterization of abundant FFA species in the skeletal muscle of obese individuals may become a useful tool to predict the progression of sarcopenic obesity.

**Abstract:**

Age-related loss of skeletal muscle is associated with obesity and inflammation. In animal models, intramuscular fat deposits compromise muscle integrity; however, the relevant fat components that mediate muscular inflammation are not known. Previously, we hypothesized that free fatty acids (FFAs) may directly induce inflammatory gene expression in skeletal muscle cells of obese rats. Here, we examined this hypothesis in primary human skeletal myoblasts (SkMs) using multiplex expression analysis of 39 inflammatory proteins in response to different FFA species. Multiplex mRNA quantification confirmed that the *IL6*, *IL1RA*, *IL4*, *LIF*, *CXCL8*, *CXCL1*, *CXCL12* and *CCL2* genes were differentially regulated by saturated and unsaturated C16 or C18 FFAs. Fluorescence staining revealed that only saturated C16 and C18 strongly interfere with myoblast replication independent of desmin expression, mitochondrial abundance and oxidative activity. Furthermore, we addressed the possible implications of 71 human receptor tyrosine kinases (RTKs) in FFA-mediated effects. Phosphorylated EphB6 and TNK2 were associated with impaired myoblast replication by saturated C16 and C18 FFAs. Our data suggest that abundant FFA species in human skeletal muscle tissue may play a decisive role in the progression of sarcopenic obesity by affecting inflammatory signals or myoblast replication.

## 1. Introduction

The age-related progressive loss of skeletal muscle mass and strength is frequently accompanied by excessive obesity, a condition known as sarcopenic obesity [1]. Epidemiological studies have revealed that obesity increases the prevalence of sarcopenia, suggesting a direct link between the excessive accumulation of fat in muscle tissue and muscle degeneration [2,3]. In animal models, increased intramuscular lipid accumulation was found to compromise muscle protein anabolism through lipotoxicity and cellular dysfunction [4,5].

Lipids are classified into four major ester lipid classes: cholesterol esters, triacylglycerols, glycerophospholipids (GPLs), and free fatty acids (FFAs). Excessive accumulation of FFAs in skeletal muscle has been attributed to ectopic lipid deposition resulting from enhanced lipid uptake or decreased lipid use through oxidation, lipid release and/or secretion [6,7]. Early clinical studies demonstrated that elevated levels of circulating FFAs induce insulin resistance within 2–4 h in all individuals irrespective of sex and age [8]. Immortalized rodent muscle cell lines have been employed to study the molecular mechanisms of FFA actions in the regulation of metabolism, apoptosis and insulin resistance [9,10,11,12]. To extend the relevance of the outcomes to humans, some studies have utilized in vitro differentiated human myotubes to study the effects of FFAs on lipid metabolism, mitochondrial function and the expression of particular genes of interest [13,14,15,16]. In organisms, FFAs commonly exhibit diverse primary or secondary structures. Common informal names of fatty acids originate from their natural sources, highlighting their function as energy supplies or nutrients. However, recent studies in macrophages, adipocytes and endothelial cells have shown that FFAs act as signaling molecules and that their chemical characteristics play a decisive role in signal transduction and gene activation [17,18,19,20].

Inflammation has been implicated in skeletal muscle wasting in animal studies [21,22,23]. The high level of systemic inflammatory proteins was suggested to play a key role in the development of human sarcopenic obesity; however, it has been difficult to clarify which proteins are clinically relevant [21,22,24]. One limitation of clinical studies is the reverse causality mechanism, implying that sarcopenic obesity could be a risk factor for the development of an inflammatory state. Moreover, inflammation involves a complex network of numerous interacting pro- and anti-inflammatory proteins and their receptor proteins that modulate the magnitude and dynamics of inflammation. Despite numerous other naming systems, a unified nomenclature of encoding genes was established that refers to their role as an interleukin (IL), the arrangement of N-terminal cysteine residues (CC or CXC) or their function as a receptor (R) or ligand protein (L) (https://www.genenames.org/, accessed on 6 May 2021).

Skeletal muscle tissue regeneration is facilitated by adult stem cells, named satellite cells (SCs). SCs proliferate to expand the population of their progeny, named myoblasts, which are capable of replication, as well as final fusion to form myotubes and myofibers. Defects in myoblast functions can result in a loss of muscle mass and decline in performance, which are characteristics of patients with sarcopenia. The immortalized murine cell line C2C12 or transformed rat cell line L6 is commonly utilized to study the regulation of metabolism and myogenesis in skeletal muscle cells [9,10,11,12,25,26,27]. Few studies have utilized in vitro differentiated human myotubes obtained by long-term maintenance of human myoblasts from multiple donors in the presence of recombinant growth hormones [13,14,15,16]. A comparative study revealed that in vitro differentiated human myotubes deviate significantly from their precursor myocytes [28]. Here, we utilized undifferentiated myoblasts from a single healthy young donor to avoid functional alterations of cells or confounding results that may arise from the genetic and metabolic diversity of donors. The objective of our study was to establish a comprehensive inflammatory expression profile of primary human skeletal myoblasts and to determine the relevance of different FFA configurations for the activation of cellular pathways.

## 2. Materials and Methods

### 2.1. Fatty Acids (FAs)

Analytical-grade fatty acids (FAs) were obtained from Biotrend Chemikalien GmbH, Cologne, Germany (1008, 1010, 1014, 1020, 1022, 1024, 1149, 1151, 1208, HYB0660). FAs were conjugated with bovine serum albumin (BSA) at a 1:2.5 ratio, as previously recommended [29]. Briefly, 6 mM FAs and 2.4 mM FA-free BSA (PAN Biotech, Aidenbach, Germany) were dissolved in water and incubated at 50 °C for 5 min. An equivalent FA-free BSA solution was prepared for control experiments.

### 2.2. Cell Culture

Primary human skeletal muscle myoblasts (SkMs) were obtained from Lonza, Basel, Switzerland (CC-2561). All cell stocks in this report were derived from the same nineteen-year-old healthy male donor and were subjected to experiments at the 7th doubling cycle. All cells tested positive for MyoD and negative for mycoplasma, bacteria, yeast and fungi. SkMs were maintained in SkGM2 BulletKit Medium (Lonza, Basel, Switzerland CC-3245) at 37 °C and 5% CO_2_, according to the manufacturer’s instructions. For stimulation, SkMs were seeded (6200 cells/cm^2^) and maintained in SkGM2 BulletKit Medium (CC-3245, Lonza) for 48 h. SkMs were then incubated in SkGM2 BulletKit Medium with equal amounts of mock or FA solution (0.05 mM final concentration of FFAs) for 24 h for mRNA quantification or 48 h for protein quantification.

### 2.3. Multiplex Protein Quantification

Human Cytokine & Chemokine 34-Plex (EPXR340-12167-901) and customized ProcartaPlex immunoassays were based on antibody-coated magnetic beads and obtained from Thermo Fisher Scientific (Waltham, MA, USA). Sample preparation and analysis were performed using Luminex xMAP technology-based Magpix (Thermo Fisher Scientific, Waltham, MA, USA) according to the manufacturer’s instructions. Briefly, SkM total protein extracts were prepared using Procarta Plex Cell Lysis Buffer (Thermo Fisher Scientific, Waltham, MA, USA). Total protein concentrations were determined using a Pierce 660 nm Protein Assay Kit (22662, Thermo Fisher Scientific, Waltham, MA, USA), according to the manufacturer’s instructions.

### 2.4. Multiplex mRNA Quantification

Designated mRNAs were quantified using a preconfigured Quantigene Plex assay (Thermo Fisher Scientific) based on direct hybridization to specifically designed capture extender (CE), label extender (LE) and blocking probes (BL). Sequences are provided in Supplemental Figure S1. GAPDH and GUSB mRNAs were used as internal controls for quantitative comparison of mRNAs among the samples. Sample preparation and analysis were performed using Luminex xMAP technology-based Magpix, according to the manufacturer’s instructions (Thermo Fisher Scientific, Waltham, MA, USA).

### 2.5. Monitoring Mitochondrial Abundance and Function

MitoTracker Red CMXRos, a derivative of red-fluorescent X-rosamine (M7512, Thermo Fisher Scientific, Waltham, MA, USA), and a chemically reduced form of tetramethylrosamine, MitoTracker Orange CM-H_2_TMRos (M7511, Thermo Fisher Scientific), were used to monitor all or only active mitochondria, respectively. SkMs were seeded on 96-well plates (1600 cells/cm^2^) and stimulated with FAs or left unstimulated, as described above. Before imaging, cells were incubated with 1 µM MitoTracker Orange CM-H2TMRos or 200 nM MitoTracker Red CMXRos in DMEM (P04-0359, PAN Biotech, Aidenbach, Germany) for 60 or 30 min, respectively.

### 2.6. Immunohistochemistry

After the indicated treatments, SkMs were seeded on chamber slides (3500 cells/cm^2^). After 24 h, the supernatant was removed, and the cells were washed with PBS (PAN Biotech, Aidenbach, Germany), covered with 4% formaldehyde solution (27248, Otto Fischar GmbH, Saarbrücken, Germany) for 10 min and rinsed with 0.1% Tween 20 (9127.1, Carl Roth GmbH, Karlsruhe, Germany) in PBS 3 times. Fixed SkMs were permeabilized for 10 min using 0.1% Triton X-100 (T8787, Merck KGaA, Darmstadt, Deutschland) in PBS and incubated with 1:200 diluted skeletal muscle actin antibody (MA5-12542, Thermo Fisher Scientific, Waltham, MA, USA) and the VectaFluor Amplified Kit (DK2488, Vector Laboratories, Burlingame, CA, USA) or with 1:200 diluted desmin antibody (ab227651, Abcam, Cambridge, UK) and the VectaFluor Amplified Kit (DK1594, Vector Laboratories) for staining, according to the manufacturer’s protocol. After staining, cells were covered with a coverslip in mounting medium with DAPI (DK2488, Vector Laboratories).

### 2.7. Cellular Lipid Storage

Lipophilic green-fluorescent BODIPY 493/503 (D3922, Thermo Fisher Scientific, Waltham, MA, USA) was used for lipid staining. SkMs were seeded at a density of 1000 cells/cm^2^ and treated as indicated. After 48 h, the cells were carefully washed in PBS (PAN Biotech, Aidenbach, Germany) and incubated with 2 µM BODIPY (5 mM in DMSO) in serum-free DMEM (PAN: P04-03590) for 30 min at 37 °C. Cells were washed again in PBS (PAN Biotech) before imaging.

### 2.8. Fluorescence Microscopy/Imaging

After immunostaining, images of cells were captured using an automated upright microscope (DM6000B, Leica Microsystems, Wetzlar Germany) with a 340–380 nm filter for DAPI, 450–490 nm filter for actin or 590 nm filter for desmin. After MitoTracker staining, cell images were captured using an automated inverted microscope (DM4000B, Leica Microsystems) and 515–560 nm filter. Processing and analysis of cell images were performed using ImageJ software (https://imagej.nih.gov/ij/, accessed on 6 June 2021).

### 2.9. Protein Phosphorylation Array

Human RTK Phosphorylation Antibody Array 1 (AAH-PRTK-G1-4, RayBiotech Life, Peachtree Corners, GA, USA) was used for simultaneous identification of the relative levels of phosphorylation of 71 different human receptor tyrosine kinases (RTKs) in SkMs. Following the indicated treatment, the supernatant was removed, and SkMs were washed in cold PBS twice and solubilized in Cell Lysis Buffer with protease and phosphatase inhibitors, according to the manufacturer’s instructions. Total protein concentrations were determined as described above, and each array was incubated with 20 µg of total protein at 4 °C overnight. After subsequent wash steps, arrays were scanned using a laser scanner (GenePix 4000), and the obtained array images were analyzed using GenePixPro 7 Microarray Acquisition and Analysis Software (Molecular Devices, San Jose, CA, USA).

### 2.10. Statistical Analysis

All results are reported as the relative means of values normalized to the untreated control  ±  standard deviations. The results were compared using ANOVA multiple comparison testing with Bonferroni’s multiple comparisons. The alpha level was considered statistically significant at *p* ≤ 0.05 (*).

## 3. Results

### 3.1. FFAs Affect the Expression of Eight Inflammatory Proteins in Primary Human Myoblasts

Previously, we reported a strong abundance of FFAs and inflammatory proteins in the skeletal muscle of aging sarcopenic rats, indicating a key role of FFAs in the regulation of skeletal muscle inflammation [30]. We hypothesized that FFAs would analogously be involved in the pathogenesis of sarcopenia in the human system. To examine this hypothesis, we utilized primary human SkMs, which play an important role in maintaining skeletal muscle mass. Despite a limited lifespan, primary cells are known to retain their function and a phenotype close to that of the source tissue. Furthermore, the application of identical passages of SkMs from a single healthy adult together with analytical grade purified FFAs allowed us to make a direct comparison of data obtained from the entire study. In the present study, we simplified the nomenclature of FFAs to provide all relevant information on chemical structures. The number of carbon atoms is depicted first, followed by square brackets enclosing the number of unsaturated carbon double bonds and the letter “c” or “t”, signifying the cis or trans configuration, respectively.

**Figure 1:**
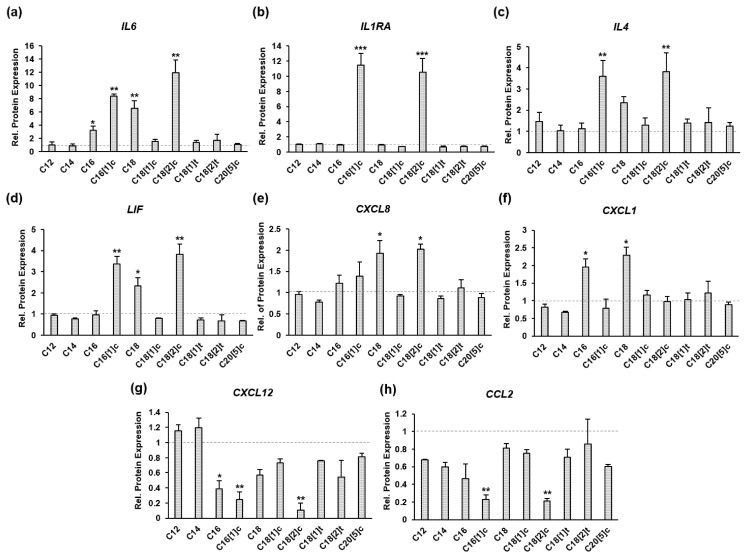
FFAs regulate the expression of inflammatory proteins in human primary myocytes. SkMs were treated with FAs conjugated with BSA or BSA alone for 48 h in three independent sets of experiments. SkM cell extracts were subjected to multiplex protein quantification three times to precisely determine the concentration of inflammatory markers in total cellular proteins. For a direct comparison of effects, the level of each marker in FFA-treated cells was compared to its expression level in untreated cells to obtain the relative protein expression (**a**–**h**). Thus, the level of expression in untreated control cells was set to 1 (dashed gray line). The results are presented as the mean ± SD from three independent experiments. Gray bars indicate the relative expression of protein markers indicated at the top of each diagram. Statistical significance was calculated using one-way ANOVA with Bonferroni’s multiple comparison test. *p* ≤ 0.05 (*). *p* ≤ 0.01 (**). *p* ≤ 0.005 (***).

SkMs were treated with different FFAs conjugated with BSA or BSA alone (control) for 48 h in three independent sets of experiments under identical conditions. All SkM extracts were subjected to a multiplex inflammation detection panel that allowed simultaneous quantification of 39 inflammatory markers. All samples were analyzed in three parallel assays to determine the concentration of inflammatory markers in total cellular proteins. For a direct comparison of effects, we normalized the level of each protein marker in FFA-treated cells to its expression level in control cells. The result is shown as the relative protein expression (Figure 1a–h). Thus, the level of expression in untreated control cells was set to 1 (dashed gray line). IL-6, IL-1RA, IL-4, LIF, CXCL8, CXCL1, CXCL12 and CCL2 were differentially regulated in response to distinct FFAs (Figure 1a–h). Interestingly, IL-6, IL-1RA, IL-4, LIF, CXCL8 and CXCL1 levels were elevated in contrast to CXCL12 and CCL, which were inhibited in response to C16[1]c and C18[2]c. The IL-6 and IL1RA genes showed the highest level of induction, which was up to 13-fold higher than that of control cells (Figure 1a,b). In general, C16 and C18 FFAs revealed the most prominent effects; however, the C18[1]c and trans configurations of C18[1]t and C18[2]t showed no detectable effects (Figure 1a–h). The data suggested a potential role of distinct FFAs in skeletal muscle inflammation and identified IL-6, IL-1RA, IL-4, LIF, CXCL8, CXCL1, CXCL12 and CCL2 as relevant inflammatory markers in human primary myoblasts.

### 3.2. FFAs Regulate Inflammatory Target Genes at the Transcriptional Level

**Figure 2:**
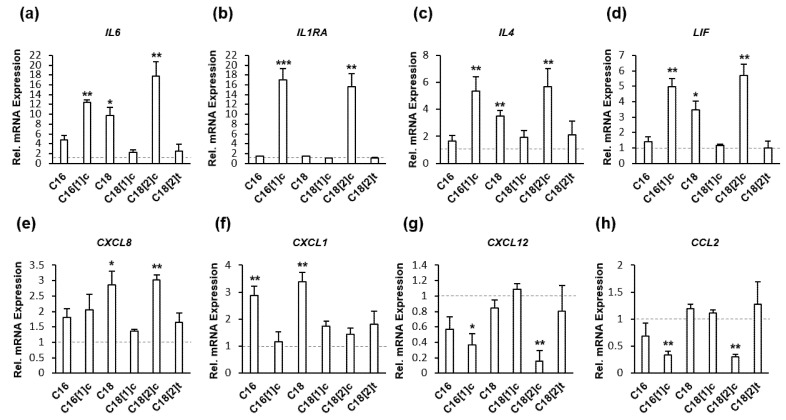
FFAs regulate the transcriptional activity of inflammatory genes in human primary myocytes. SkMs were treated with FAs conjugated with BSA or BSA alone for 24 h in three independent sets of experiments. SkM cell extracts were subjected to multiplex mRNA quantification three times to determine the concentration of designated mRNAs in the total cellular RNA. For a direct comparison of effects, the expression of each mRNA in FFA-treated cells was compared to its expression level in untreated cells to obtain the relative fold change in expression (**a**–**h**). Thus, the expression level in untreated control cells was set to 1 (dashed gray line). The results are presented as the mean ± SD of the relative expression of designated markers in 3 independent experiments (white bars). Statistical significance was calculated using one-way ANOVA with Bonferroni’s multiple comparison test. *p* ≤ 0.05 (*). *p* ≤ 0.01 (**). *p* ≤ 0.005 (***).

Next, we determined the abundance of IL4, IL6, CXCL8, IL1RA, CCL2, LIF, CXCL1 and CXCL12 mRNAs in SkMs using a direct multiplex approach that eliminates possible inconsistencies due to cDNA synthesis or amplification reactions. All experiments were performed under identical conditions, as described above (Section 3.1). We performed three independent experiments and determined the abundance of encoding mRNAs among the total amount of cellular mRNA using two housekeeping genes. For a direct comparison of effects, we normalized the expression of each mRNA in FFA-treated cells to its expression in control cells. Thus, the level of expression in untreated control cells was set to 1 (dashed gray line). As shown in Figure 2a–h, the IL4, IL6, CXCL8, IL1RA, CCL2, LIF, CXCL1 and CXCL12 genes were regulated at the transcriptional level. The upregulation and downregulation rates of all mRNAs corresponded to the level of protein expression depicted in Figure 1a–h. We suggest that the C16, C16[1]c, C18 and C18[2]c FFAs are implicated in the activation of diverse signal transduction pathways that differentially orchestrate and fine-tune the extent of inflammatory gene transcription.

### 3.3. Saturated C16 and C18 Inhibit Human Skeletal Myoblast Expansion

**Figure 3:**
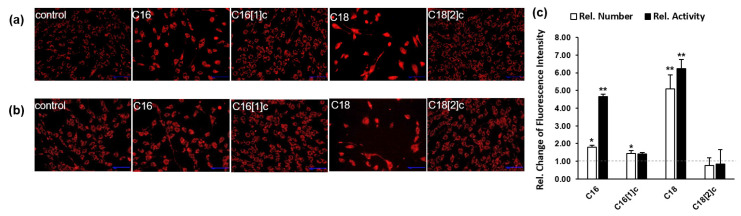
Mitochondrial abundance and activity in human primary myoblasts were differentially affected by FFAs. SkMs were treated with FAs conjugated with BSA or BSA alone (control). After 48 h, SkMs were incubated with MitoTracker Red (**a**) or Orange (**b**). Fluorescence images at identical settings and exposure times from 3 sets of experiments were simultaneously imported into the analysis software, processed to 8-bit images and analyzed. The integrated fluorescence intensity of FFA-treated cells was compared to that of control cells, which was set to 1 (c, dashed gray line). The results are presented as the mean ± SD of the relative fluorescence intensity of MitoTracker Red (white bars) or Orange (black bars). Statistical significance was calculated using one-way ANOVA with Bonferroni’s multiple comparison test. *p* ≤ 0.05 (*), *p* ≤ 0.01 (**).

FFA levels were associated with increased permeabilization of mitochondrial membranes and inhibition of oxidative and energy-conserving systems in hepatocytes [31]. To address the possible effects of different FFAs on mitochondrial abundance in SkMs, we used a derivative of red-fluorescent X-rosamine, MitoTracker Red, to detect the number of active mitochondria in SkMs. This dye is a chemically reduced form of tetramethylrosamine and accumulates in active mitochondria (Figure 3a). In a parallel set of experiments, we used MitoTracker Orange to monitor potential alterations in mitochondrial activity. This dye is a nonfluorescent tetramethylrosamine that accumulates in mitochondria and excites orange fluorescence depending on the activity of the oxidative system (Figure 3b). Treatments with saturated C16 and C18 FFAs revealed relatively low numbers of enlarged SkMs within 48 h (Figure 3a,b). Notably, we did not observe an increase in the number of detached or dead SkMs, indicating that C16 and C18 FFAs possibly interfered with the expansion of cells. Full-size images from three independent sets of experiments were processed and analyzed to obtain the amount (Figure 3a) and intensity of fluorescent signals (Figure 3b). For a direct comparison of effects, the quantity and activity of mitochondria in FFA-treated cells were compared to the data in control cells, which were thereby set to 1 (dashed gray line). The relative number of mitochondria was substantially increased by treatment of SkMs with saturated FFA C18 (Figure 3c). The relative mitochondrial activity was strongly elevated by both saturated C16 and C18 FFAs (Figure 3c).

### 3.4. Desmin Expression in Human Primary Myoblasts Is Not Affected by FFAs

**Figure 4:**
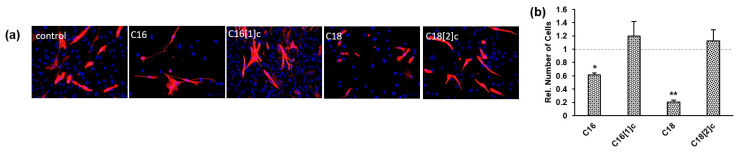
FFAs do not affect the desmin expression pattern in human primary myoblasts. (**a**) SkMs were treated with FAs conjugated with BSA or BSA alone (control) before DAPI and desmin staining. (**b**) Fluorescence images from 3 sets of experiments were analyzed, as described in the legend to Figure 3. The number of DAPI-positive FFA-treated cells was compared to that of control cells, which was set to 1 (c, dashed gray line). The data are presented as the mean ± SD of cell number (dotted bars). Statistical significance was calculated using one-way ANOVA with Bonferroni’s multiple comparison test. *p* ≤ 0.05 (*). *p* ≤ 0.01 (**).

Undifferentiated skeletal myoblasts temporarily express desmin, which plays an important role in maintaining muscle mass and function. We examined whether FFAs may alter the desmin expression pattern in SkMs using immunofluorescence staining, and DAPI was used as a nuclear counterstain. As shown in Figure 4a, control SkMs expressed desmin at different levels. We found that 10–15% of SkMs accumulated desmin at the highest levels, which overshadowed the signal of low-expressing cells. Importantly, FFAs did not affect the characteristic pattern of desmin expression in SkMs. DAPI images were processed and analyzed to obtain the number of SkMs in three independent sets of experiments. Figure 4b shows the relative number of cells among FFA-treated cells compared to the number of control cells, which was set to 1 (dashed gray line). As suggested through mitochondrial assessments (Section 3.3), the number of cells was reduced by saturated C16 and C18. Again, we observed no significant increase in dead cells in the culture, and the overall structure and size of the nucleus were indistinguishable among the different samples.

### 3.5. C18[2]c Increases Lipid Accumulation in Human Primary Myoblasts

**Figure 5:**
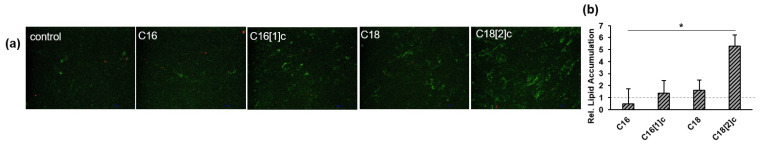
FFAs are differentially utilized in human primary myoblasts. (**a**) SkMs were treated with FAs conjugated with BSA or BSA alone (control) for 48 h and stained using BODIPY. (**b**) Fluorescence images from 3 sets of experiments were analyzed as described in the legend to Figure 3. The fluorescence intensity of FFA-treated cells was compared to that of control cells, which was set to 1 (c, dashed gray line). The results are presented as the mean ± SD of relative lipid accumulation (hatched gray bars). Statistical significance was calculated using one-way ANOVA with Bonferroni’s multiple comparison test. *p* ≤ 0.05 (*).

Skeletal muscle cells are able to store FFAs such as triacylglycerol in lipid droplets, a process that may lead to lipotoxic stress and inflammation [32,33]. To examine possible differences in the utilization of FFAs, we used a green-fluorescent dye with hydrophobic properties for staining lipids and other lipophilic compounds. In three independent sets of experiments, SkMs were treated with different FFAs or control for 48 h prior to staining. As shown in Figure 5a, we observed increased accumulation of lipids in SkMs that were treated with double-unsaturated C18 (Figure 5a). Next, all images were processed and analyzed simultaneously. We captured the overall intensity of all signals in each sample to estimate lipid accumulation. The levels in FFA-treated cells were compared to those in control-treated cells to determine the relative accumulation of lipids. We observed a significant increase in lipid droplets exclusively in C18[2]c-treated SkMs (Figure 5b).

### 3.6. FFAs Activate Different Receptor Tyrosine Kinases in Human Primary Myoblasts

**Figure 6:**
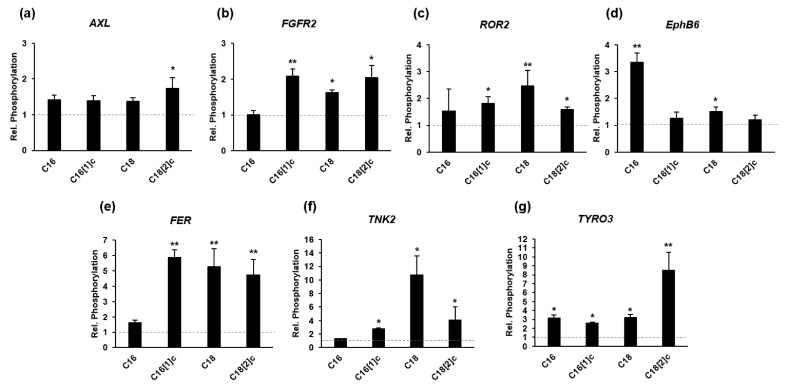
FFAs selectively activate RTK phosphorylation in human primary myoblasts. SkMs were treated with FAs conjugated with BSA or BSA alone for two hours. Whole-cell lysates were prepared and each incubated with a single RTK array. Following incubation steps, the slides were washed, imaged and analyzed using a laser scanner. Fluorescence images were processed and analyzed simultaneously using analysis software. To obtain the relative phosphorylation (black bars) of individual RTKs indicated at the top of each diagram, integrated signal intensities from FFA-treated cells were compared to that from control cells, which was set to 1 (**a**–**g**, dashed gray line). Data are presented as the mean ± SD of the relative phosphorylation. Statistical significance was calculated using one-way ANOVA with Bonferroni’s multiple comparison test. *p* ≤ 0.05 (*). *p* ≤ 0.01 (**).

Few members of the RTKs have been implicated in FFA-mediated insulin resistance, cell growth and development [34,35,36]. This prompted us to identify RTKs as possible targets of FFAs in SkMs. We utilized preconfigured triplicate arrays of antibodies against 71 human RTKs as well as triplicates of four positive and four negative controls. Arrays were incubated with whole-cell lysates from SkMs that were treated with C16, C16[1]c, C18, C18[2]c or BSA for two hours. Following incubation with biotinylated anti-phosphotyrosine antibodies and fluorescent-conjugated antibodies, the slides were scanned and analyzed. Before further comparison, the raw intensity data were processed, yielding a high-quality normalization by background correction, log transformation and normalization relative to positive controls. The estimated fluorescence intensity of RTKs in FFA-treated SkMs was compared to the corresponding RTK signal intensity in control cells to obtain the relative phosphorylation. We observed significant differences in the phosphorylation of only 7 RTKs (Figure 6a–g). Phosphorylated *TYRO3* and *FER* were most significantly elevated by C16, C16[1]c, C18 and C18[2]c, which affected the inflammatory gene expression (Figure 6e,g). This was also observed by *AXL*, *FGFR2* and *ROR2* phosphorylation, though to a lower extent. Saturated C16 and C18 led to the highest levels of phosphorylated *EphB6* and *TNK2*, respectively. We suggest that these RTKs may be involved in the inhibitory effects of C16 and C18 on SkM replication.

## 4. Discussion

The pathogenesis of sarcopenic obesity is complex and involves multiple interacting factors, such as age-related changes in body composition and physical activity. The decrease in muscle mass can accelerate the infiltration of adipose cells in muscle tissue, reducing contraction efficiency and muscle strength, which may also lead to a decrease in physical activity [2]. In addition, hypertrophic adipocytes can accumulate lipids and release FFAs in skeletal muscle tissue. The main finding of this study was that primary human skeletal myoblasts act as potent producers of inflammatory proteins by direct response to distinct species of FFAs. Discrete structural characteristics of FFAs were identified to be relevant to the inflammatory gene response or impaired replication of human skeletal myoblasts through activation of receptor tyrosine kinases. Taken together, the results of this study provide the first evidence that FFAs induce inflammatory markers that potentially activate tissue-resident immune cells, e.g., macrophages; contribute to the inflammatory status in human skeletal muscle tissue; and significantly restrain muscle regeneration.

One possible limitation of our study may be the functional deviation of human myoblasts after isolation from source tissue. However, primary cells were expected to preserve the functional characteristics of the source tissue compared to excessively cultivated, differentiated or immortalized cells. Moreover, the primary myoblasts in our study were used at the seventh doubling cycle, which corresponds to 5.25 days ex vivo, and are therefore less likely to have deviated from tissue resident myoblasts. Thus, the application of SkMs is a potentially more predictive strategy for the study of FFA-mediated effects in human skeletal muscle. A comparative study of several in vitro differentiated human myotubes and immortalized cell lines revealed significant differences in gene expression and cellular morphology [28]. In agreement with these observations, we detected 13 constitutively expressed inflammatory proteins in primary human myoblasts (Supplement Figure S2). However, none of these genes were expressed in the immortalized human muscle cell line TE-671 (data not shown).

Primary human skeletal myoblasts have not yet been studied with respect to their inflammatory response to FFAs. Our data indicated that FFAs shorter than C16 or longer than C18 or those in the *trans* configuration had no effects on inflammatory gene expression in human myoblasts. Only C16, C18, C16[1]c and C18[2]c affected gene expression, emphasizing the relevance of chain length and steric configuration in FFA-mediated signaling. Interestingly, C18[1]c was the only exception that did not affect inflammatory gene expression (Figure 1 and Figure 2) or mitochondria, desmin expression, lipid accumulation or RTK phosphorylation (data not shown). Therefore, structural properties other than chain length, saturation or *cis* configuration likely play an additional role in the regulation of primary human myoblasts. It is important to note that our study monitored the inflammatory response to different species of FFAs individually. However, the abundant combination of FFAs in vivo may act differentially or synergistically. Furthermore, we focused on a selected group of inflammatory markers based on the response of a single donor. Future studies will be needed to verify our data in myoblasts from different donors because environmental and epigenetic factors are known to strongly affect the extent of the inflammatory gene response [37].

It has been previously suggested that FFAs may affect lipid metabolism in differentiated human myotubes [14,15]. In human myoblasts, C18[2]c exclusively led to lipid accumulation within 48 h. Although the long-term effects of C18[2]c were not studied here, we assume that unceasing lipid accumulation may affect signaling pathways and gene expression. Nevertheless, the reported effects of FFAs on RTK phosphorylation after two hours and on gene expression after 24 h are unlikely to have been a consequence of lipid accumulation. Another limitation of this study may be that the presented results referred to a single time point after treatment, rather than reflecting the dynamics of FFA-mediated actions. We note that FFA exposure times of one and three hours had negligible effects on RTK phosphorylation, mitochondrial performance and lipid accumulation (data not shown). However, further detailed studies are required to uncover possible differences in the action dynamics of various FFAs.

The FFAs C16, C18, C16[1]c and C18[2]c differentially regulated the *IL6*, *IL1RA*, *IL4*, *LIF*, *CXCL8*, *CXCL1*, *CXCL12* and *CCL2* genes in primary human myoblasts. These genes are likely relevant to the role of myoblasts specifically in FFA-mediated skeletal muscle inflammation, since 31 inflammatory genes were not affected in response to FFAs (Supplemental Figure S3). As expected, many but not all protein markers were secreted into SKM medium simultaneously (data not shown). However, a comparative analysis of the amount of secreted individual markers in medium was challenging because of potential variations in supernatant volumes and the lack of suitable normalization. Thus, our data demonstrated the steady state of protein and mRNA expression in SkMs (Figure 1 and Figure 2). *IL6* protein and mRNA expression was significantly induced by saturated and unsaturated FFAs in primary myoblasts, revealing the broadest responses to FFAs in our study. In a previous study using in vitro differentiated myotubes, however, saturated C16 and C18 were selectively able to activate the IL-6 gene [13]. Despite conceivable differences between in vitro and in vivo differentiated myotubes, it is possible that the *IL6* response to unsaturated FFAs was abolished during myogenic differentiation. Nevertheless, the saturation of FFAs may play only a minor role in the induction of the *IL6* gene in primary myoblasts.

Here, for the first time, *IL1RA* was identified as an FFA target gene in primary human skeletal myoblasts. Myeloid cells, hepatocytes, fibroblasts and mesenchymal stem cells were found to express *IL1RA*, which acted as an important mediator of inflammation and tissue damage [38,39]. Moreover, *IL1RA* was initially identified as a natural antagonist of IL1A and IL1B by binding IL1R1, which plays a central role in sensing irregularities in the tissue that require immediate and adequate attention by the immune system [40]. Thus, C16[1]c- and C18[2]c-mediated *IL1RA* expression may modulate or prolong the response of myoblasts to IL1A and IL1B.

*IL4* and *LIF* have been previously implicated in the activation of myotube fusion [41,42]. In addition, *CXCL12* and *CCL2* were previously recognized to play important roles in the development and differentiation of progenitor satellite cells [43,44]. Our data indicated that C16[1]c, C18 and C18[2]c increase *IL4* and *LIF* and decrease *CXCL12* and *CCL2* expression in myoblasts. Thus, the abundance of C16[1]c, C18 and C18[2]c in skeletal muscle may potentially impede the commitment of skeletal progenitor cells to myogenesis but promote myotube formation.

Accumulation of saturated C16 in skeletal muscle has been associated with muscle atrophy and impaired replication of immortalized murine myoblasts [45,46]. Our data were consistent with these findings, showing that SkM replication was restrained by C16 but completely blocked by C18. In contrast, FFAs shorter than C16 or longer than C18 or those in the *trans* configuration had no effects on the replication of human myoblasts (data not shown). Moreover, neither the abundance and activity of mitochondria nor the synthesis of cellular proteins were affected by C16 and C18. Importantly, the equal abundance of desmin-positive SkMs negated the possible impact of C16 and C18 on primary human myoblast differentiation. Despite these parallels, C16 and C18 likely induce discrete pathways by the phosphorylation of *EphB6* and *TNK2*, respectively. EphB6 and *TNK2* have been previously implicated in signaling pathways that are relevant to cell proliferation [47,48]. EphB6 was found to regulate vascular smooth muscle contractility in mice [49]. However, further studies will be needed to specify the functions of *EphB6* and *TNK2* in skeletal muscle myoblasts and their specific responses to saturated C16 and C18.

## 5. Conclusions

Our study provides the first comprehensive analysis of primary human myoblasts and identifies distinct FFAs that trigger inflammation or inhibit cell replication in human skeletal muscle. There is a strong need for early prediction of sarcopenic obesity, a condition for which the diagnostic criteria are inconsistent and for which the risk of mortality is higher than that of obesity or sarcopenia alone. Thus, structural analysis of abundant FFA species in human skeletal muscle tissue may serve as a predictive tool for the early diagnosis of muscle loss progression in sarcopenic obesity. However, future clinical studies will be required first to compare the abundance of FFA species between healthy and sarcopenic human muscle specimens and second to prove the suitability of FFA profiling in the diagnosis of sarcopenic obesity.

## Data Availability

Data are contained within the article or supplementary material.

## Supplementary Materials

The following are available online at https://www.mdpi.com/article/10.3390/biology10121318/s1. Figure S1: Gene sequences used for Quantigene experiments. Figure S2: Constitutively expressed inflammatory proteins in primary human myoblasts. Figure S3: Expression of inflammatory proteins in human primary myocytes in response to FFAs.
