# Supplementary material for: Free Fatty Acid Species Differentially Modulate the Inflammatory Gene Response in Primary Human Skeletal Myoblasts
Source: Biology (Basel). 2021 Dec 12;10(12):1318. doi: 10.3390/biology10121318 (PMC8698660; doi:10.3390/biology10121318)
Supplement: Supplementary file 1 [file biology-10-01318-s001.zip › biology-1492887-supplementary.pdf]

(*CCL2*):
gagaggctgagactaacccagaaacatccaattctcaaactgaagctcgcactctcgcctccagcatgaaagtctctgcc
gcccttctgtgcctgctgctcatagcagccaccttcattccccaagggctcgctcagccagatgcaatcaatgccccagt
cacctgctgttataacttcaccaataggaagatctcagtgcagaggctcgcgagctatagaagaatcaccagcagcaagt
gtcccaaagaagctgtgatcttcaagaccattgtggccaaggagatctgtgctgaccccaagcagaagtgggttcaggat
tccatggaccacctggacaagcaaacccaaactccgaagacttgaacactcactccacaacccaagaatctgcagctaac
ttattttcccctagctttccccagacaccctgttttattttattataatgaattttgtttgttgatgtgaaacattatgc
cttaagtaatgttaattcttatttaagttattgatgttttaagtttatctttcatggtactagtgtttttagatacaga
gacttggggaaattgcttttcctcttgaaccacagttctacccctgggatgttttgagggtctttgcaagaatcattaat
acaaagaatttttttaacattccaatgcattgctaaaatattattgtggaaatgaatattttgtaactattacaccaaa
(*CXCL8*):
ctttaatttcaggaattgaatgggtttgctagaatgtgatatttgaagcatcacataaaaatgatgggacaataaatttt
gccataaagtcaaatttagctggaaatcctggatttttttctgttaaatctggcaaccctagtctgctagccaggatcca
caagtccttgttccactgtgccttggtttctcctttatttctaagtggaaaaagtattagccaccatcttacctcacagt
gatgttgtgaggacatgtggaagcactttaagtttttcatcataacataaattattttcaagtgtaacttattaaccta
tttattatttatgtatttatttaagcatcaaatatttgtgcaagaatttggaaaaatagaagatgaatcattgattgaat
agttataaagatgttatagtaaatttattttattttagatattaaatgatgttttattagataaatttcaatcagggttt
ttagattaaacaaacaaacaattgggtacccagttaaattttcatttcagataaacaacaaataatttttagtataagt
acattattgtttatctgaaattttaattgaactaacaatcctagtttgatactcccagtcttgtcattgccagctgtgtt
ggtagtgctgtgttgaattacggaataatgagttagaactattaaaacagccaaaactccacagtcaatattagtaattt
cttgctggttgaaacttgtttattatgtacaaatagattcttataatattatttaaatgactgcatttttaaatacaagg
ctttatatttttaactttaagatgtttttatgtgctctccaaattttttttactgtttctgattgtatggaaatataaaa
(IL4):
atcgttagcttctcctgataaactaattgcctcacattgtcactgcaaatcgacacctattaatgggtctcacctcccaa
ctgcttcccccctctgttcttcctgctagcatgtgccggcaactttgtccacggacacaagtgcgatatcaccttacagga
gatcatcaaaactttgaacagcctcacagagcagaagactctgtgcaccgagttgaccgtaacagacatctttgctgcct
ccaagaacacaactgagaaggaaaccttctgcagggctgcgactgtgctccggcagttctacagccaccatgagaaggac
actcgctgcctgggtgcgactgcacagcagttccacaggcacaagcagctgatccgattcctgaaacggctcgacaggaa
cctctggggcctggcgggcttgaattcctgtcctgtgaaggaagccaaccagagtacgttggaaaacttcttggaaaggc
taaagacgatcatgagagagaaatattcaaagtgttcgagctgaatattttaatttatgagtttttgatagctttatttt
(*IL6)*
attctgccctcgagcccaccgggaacgaaagagaagctctatctcccctccaggagcccagctatgaactccttctccac
aagcgccttcggtccagttgccttctccctggggctgctcctggtgttgcctgctgccttccctgccccagtacccccag
gagaagattccaaagatgtagccgccccacacagacagccactcacctcttcagaacgaattgacaaacaaattcggtac
atcctcgacggcatctcagccctgagaaaggagacatgtaacaagagtaacatgtgtgaaagcagcaaagaggcactggc
agaaaacaacctgaaccttccaaagatggctgaaaaagatggatgcttccaatctggattcaatgaggagacttgcctgg
tgaaaatcatcactggtcttttggagtttgaggtatacctagagtacctccagaacagatttgagagtagtgaggaacaa
gccagagctgtgcagatgagtacaaaagtcctgatccagttcctgcagaaaaaggcaaagaatctagatgcaataaccac
ccctgacccaaccacaaatgccagcctgctgacgaagctgcaggcacagaaccagtggctgcaggacatgacaactcatc
tcattctgcgcagctttaaggagttcctgcagtccagcctgagggctcttcggcaaatgtagcatgggcacctcagattg
(*LIF*)
caatgccctctttattctctattacacagcccaggggggagccgttccccaacaacctggacaagctatgtggccccaacg
tgacggacttcccgcccttccacgccaacggcacggagaaggccaagctggtggagctgtaccgcatagtcgtgtacctt
ggcacctccctgggcaacatcacccgggaccagaagatcctcaaccccagtgccctcagcctccacagcaagctcaacgc
caccgccgacatcctgcgaggcctccttagcaacgtgctgtgccgcctgtgcagcaagtaccacgtgggccatgtggacg
tgacctacggccctgacacctcgggtaaggatgtcttccagaagaagaagctgggctgtcaactcctggggaagtataag
(*CXCL12*):
gccgcactttcactctccgtcagccgcattgcccgctcggcgtccggcccccgacccgcgctcgtccgcccgcccgcccg
cccgcccgcgccatgaacgccaaggtcgtggtcgtgctggtcctcgtgctgaccgcgctctgcctcagcgacgggaagcc
cgtcagcctgagctacagatgcccatgccgattcttcgaaagccatgttgccagagccaacgtcaagcatctcaaaattc
tcaacactccaaactgtgcccttcagattgtagcccggctgaagaacaacaacagacaagtgtgcattgacccgaagcta
aagtggattcaggagtacctggagaaagctttaaacaagaggttcaagatgtgagagggtcagacgcctgaggaaccctt
catgaccctaaatcttgactacagtcaggaaaggaatcatttctatttctcctccatgggagaaaatagataagagtaga
aactgcagggaaaattatttgcataacaattcctctactaacaatcagctccttcctggagactgcccagctaaagcaat
atgcatttaaatacagtcttccatttgcaagggaaaagtctcttgtaatccgaatctctttttgctttcgaactgctagt
caagtgcgtccacgagctgtttactagggatccctcatctgtccctccgggacctggtgctgcctctacctgacactccc
ttgggctccctgtaacctcttcagaggccctcgctgccagctctgtatcaggacccagaggaaggggccagaggctcgtt
gactggctgtgtgttgggattgagtctgtgccacgtgtttgtgctgtggtgtgtcccctctgtccaggcactgagatac
(*CXCL1*):
ttcattagttttgaagattctatgttaatattttaggtgtaaaataattaagggtatgattaactctacctgcacactgt
cctattatattcattctttttgaaatgtcaaccccaagttagttcaatctggattcatatttaatttgaaggtagaatgt
tttcaaatgttctccagtcattatgttaatatttctgaggagcctgcaacatgccagccactgtgatagaggctggcgga
tccaagcaaatggccaatgagatcattgtgaaggcaggggaatgtatgtgcacatctgttttgtaactgtttagatgaat
gtcagttgttatttattgaaatgatttcacagtgtgtggtcaacatttctcatgttgaaactttaagaactaaaatgttc
taaatatcccttggacattttatgtctttcttgtaaggcatactgccttgtttaatggtagttttacagtgtttctggct
tagaacaaaggggcttaattattgatgttttcatagagaatataaaaataaagcacttatagaaaaaactcgtttgattt

(*IL1RA*):
atccatttacctgttttgccaagaatacacatggtatagatgcagcatatatccagttaatatatccagtcactaatttc
cagaagcacatgattggtatatgtgtcacgttgacagtcataattgtgtgttctgttttcatctataaaatcttcaagat
tgacattgtgctttggtacagggattcctgctatgattttctcccaataaaagcttcagatggaaagacctatgacgcat
atatactgtatccaaagactgttggggaagggtctacctctgactgtgatatttttgtgtttaaagtcttgcctgaggtc
ttggaaaaacagtgtggatataagctgttcatttatggaagggatgactacgttggggaagacattgttgaggtcattaa
tgaaaacgtaaagaaaagcagaagactgattatcattttagtcagagaaacatcaggcttcagctggctgggtggttcat
acctcactgaggaagggagacatattcttggagaactttccatctgcttgtattttccatacacatccccagccagaagt
tagtgtccgaagaccgaattttattttacagagcttgaaaactcacttcaatgaacaaagggattctccaggattccaaa
gttttgaagtcatcttagctttccacaggagggagagaacttaaaaaagcaacagtagcagggaattgatccacttctta
atgctttcctccctggcatgaccatcctgtcctttgttattatcctgcattttacgtctttggaggaacagctccctagt
ggcttcctccgtctgcaatgtcccttgcacagcccacacatgaaccatccttcccatgatgccgctcttctgtcatcccg
ctcctgctgaaacacctcccagggggctccacctgttcaggagctgaagcccatgctttcccaccagcatgtcactcccag
gaagaaatgacattttgctaggaagtgaccgagtctaggaatgcttttattcaagacaccaaattccaaacttctaaatg
ttggaattttcaaaaattgtgtttagattttatgaaaaactcttctactttcatctattctttccctagaggcaaacatt
tcttaaaatgtttcattttcattaaaaatgaaagccaaatttatatgccaccgattgcaggacacaagcacagttttaag
agttgtatgaacatggagaggacttttggtttttatatttctcgtatttaatatgggtgaacaccaacttttatttggaa
taataattttcctcctaaacaaaaacacattgagtttaagtctctgactcttgcctttccacctgctttctcctgggccc
gctttgcctgcttgaaggaacagtgctgttctggagctgctgttccaacagacagggcctagctttcatttgacacacag
actacagccagaagcccatggagcagggatgtcacgtcttgaaaagcctattagatgttttacaaatttaattttgcaga

(*GUSB*)
atcaccgtcaccaccagcgtggagcaagacagtgggctggtgaattaccagatctctgtcaagggcagtaacctgttcaa
gttggaagtgcgtcttttggatgcagaaaacaaagtcgtggcgaatgggactgggacccagggccaacttaaggtgccag
gtgtcagcctctggtggccgtacctgatgcacgaacgccctgcctatctgtattcattggaggtgcagctgactgcacag
acgtcactggggcctgtgtctgacttctacacactccctgtggggatccgcactgtggctgtcaccaagagccagttcct
catcaatgggaaacctttctatttccacggtgtcaacaagcatgaggatgcggacatccgagggaagggcttcgactggc
cgctgctggtgaaggacttcaacctgcttcgctggcttggtgccaacgctttccgtaccagccactacccctatgcagag
gaagtgatgcagatgtgtgaccgctatgggattgtggtcatcgatgagtgtcccggcgtgggcctggcgctgccgcagtt
(*GAPDH*):
ggaaggtgaaggtcggagtcaacggatttggtcgtattgggcgcctggtcaccagggctgcttttaactctggtaaagtg
gatattgttgccatcaatgaccccttcattgacctcaactacatggtttacatgttccaatatgattccacccatggcaa
attccatggcaccgtcaaggctgagaacgggaagcttgtcatcaatggaaatcccatcaccatcttccaggagcgagatc
cctccaaaatcaagtggggcgatgctggcgctgagtacgtcgtggagtccactggcgtcttcaccaccatggagaaggct
ggggctcatttgcaggggggagccaaaagggtcatcatctctgccccctctgctgatgcccccatgttcgtcatgggtgt
gaaccatgagaagtatgacaacagcctcaagatcatcagcaatgcctcctgcaccaccaactgcttagcacccctggcca
aggtcatccatgacaactttggtatcgtggaaggactcatgaccacagtccatgccatcactgccacccagaagactgtg
gatggcccctccgggaaactgtggcgtgatggccgcggggctctccagaacatcatccctgcctctactggcgctgccaa
aacca

Figure S1: Gene sequences are shown. Sequences of capture extenders (blue), label extenders (red), blocking probes (green) and flanking sequences (black) are indicated.

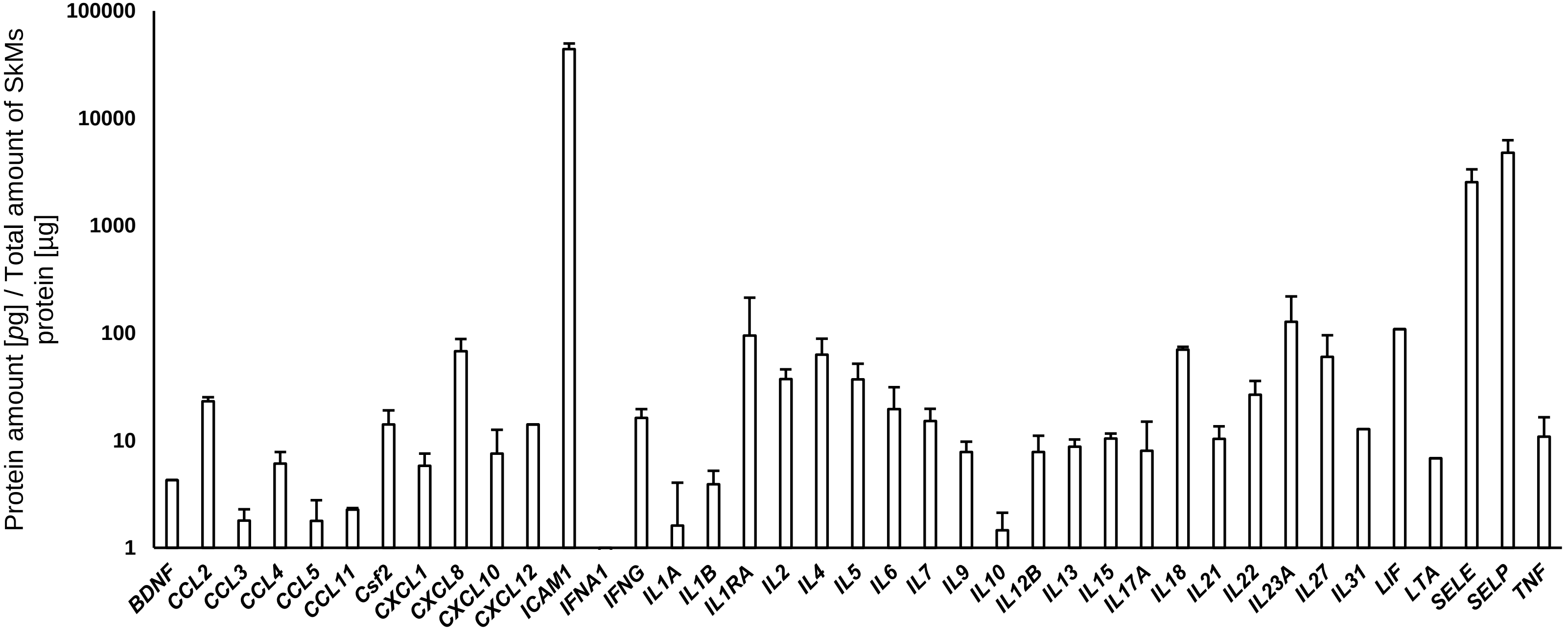

Figure S2: Constitutive expression of proteins in untreated SkMs. SkM cell extracts were subjected to multiplex protein quantification three times to precisely determine the concentration of inflammatory markers in total cellular proteins. The results are presented as the mean ± SD from three independent experiments.

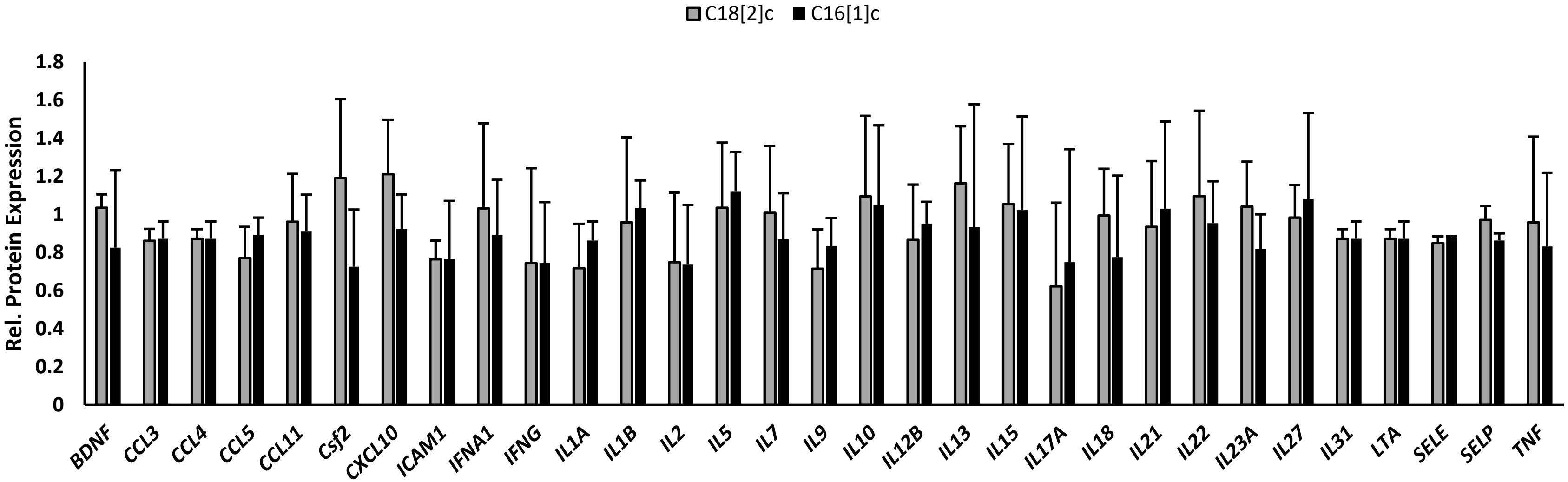

Figure S3: CC18[2]c and C16[1]c mediated relative protein expression in SkMs. The experiments and data analysis were performed as described in the legend to Fig. 1a-h.

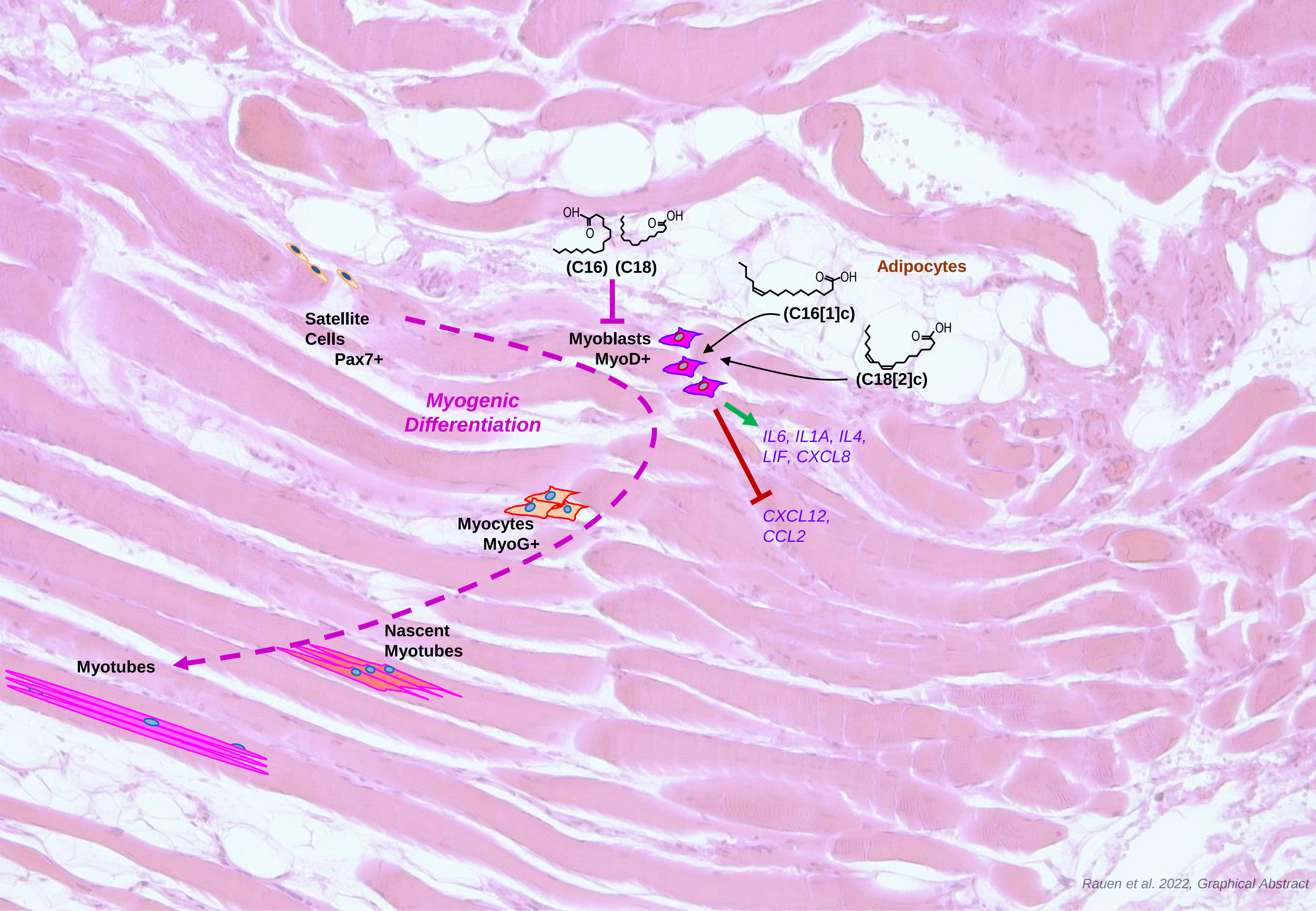
OH
O
O
OH
(C16) (C18)
Adipocytes
O
OH
(C16[1]c)
O
OH
(C18[2]c)
Satellite
Cells
Pax7+
Myoblasts
MyoD+
Myogenic
Differentiation
IL6, IL1A, IL4,
LIF, CXCL8
CXCL12,
CCL2
Myocytes
MyoG+
Nascent
Myotubes
Myotubes
Rauen et al. 2022, Graphical Abstract
